# Functional Network Alterations as Markers for Predicting the Treatment Outcome of Cathodal Transcranial Direct Current Stimulation in Focal Epilepsy

**DOI:** 10.3389/fnhum.2021.637071

**Published:** 2021-03-17

**Authors:** Jiaxin Hao, Wenyi Luo, Yuhai Xie, Yu Feng, Wei Sun, Weifeng Peng, Jun Zhao, Puming Zhang, Jing Ding, Xin Wang

**Affiliations:** ^1^School of Biomedical Engineering, Shanghai Jiao Tong University, Shanghai, China; ^2^Department of Neurology, Zhongshan Hospital, Fudan University, Shanghai, China; ^3^Department of Radiology, Zhongshan Hospital, Fudan University, Shanghai, China; ^4^CAS Center for Excellence in Brain Science and Intelligence Technology, Shanghai, China; ^5^State Key Laboratory of Medical Neurobiology, the Institutes of Brain Science and the Collaborative Innovation Center for Brain Science, Fudan University, Shanghai, China

**Keywords:** epilepsy, transcranial direct current stimulation, fMRI, functional network, graph theoretical analysis

## Abstract

**Background and Purpose:**

Transcranial direct current stimulation (tDCS) is an emerging non-invasive neuromodulation technique for focal epilepsy. Because epilepsy is a disease affecting the brain network, our study was aimed to evaluate and predict the treatment outcome of cathodal tDCS (ctDCS) by analyzing the ctDCS-induced functional network alterations.

**Methods:**

Either the active 5-day, −1.0 mA, 20-min ctDCS or sham ctDCS targeting at the most active interictal epileptiform discharge regions was applied to 27 subjects suffering from focal epilepsy. The functional networks before and after ctDCS were compared employing graph theoretical analysis based on the functional magnetic resonance imaging (fMRI) data. A support vector machine (SVM) prediction model was built to predict the treatment outcome of ctDCS using the graph theoretical measures as markers.

**Results:**

Our results revealed that the mean clustering coefficient and the global efficiency decreased significantly, as well as the characteristic path length and the mean shortest path length at the stimulation sites in the fMRI functional networks increased significantly after ctDCS only for the patients with response to the active ctDCS (at least 20% reduction rate of seizure frequency). Our prediction model achieved the mean prediction accuracy of 68.3% (mean sensitivity: 70.0%; mean specificity: 67.5%) after the nested cross validation. The mean area under the receiver operating curve was 0.75, which showed good prediction performance.

**Conclusion:**

The study demonstrated that the response to ctDCS was related to the topological alterations in the functional networks of epilepsy patients detected by fMRI. The graph theoretical measures were promising for clinical prediction of ctDCS treatment outcome.

## Introduction

Transcranial direct current stimulation (tDCS) is an emerging non-invasive brain stimulation technique that modulates the cortical excitability via the application of the constant direct currents to the scalp of subjects through two electrodes (anode and cathode) (San-juan et al., 2015). Anodal tDCS (atDCS) has been demonstrated to enhance the cortical excitability, and cathodal tDCS (ctDCS) suppresses it (Nitsche and Paulus, 2000). Since the cortical excitability is abnormally increased in epilepsy, ctDCS has been proposed as an alternative therapy for epilepsy (San-juan et al., 2015; Gschwind and Seeck, 2016). However, the treatment outcome of ctDCS is conflicting (San-juan et al., 2015). Some studies found that there was a significant reduction in seizure frequency after ctDCS treatment (Auvichayapat et al., 2013; Tekturk et al., 2016; San-Juan et al., 2017), but other studies reported negative results (Varga et al., 2011; Liu et al., 2016). The results are inconsistent, maybe because the disease entities of the patients are heterogeneous, and the therapy parameters are different (San-juan et al., 2015). Considering whether patients respond to ctDCS is not sure, early prediction of the treatment outcome is highly valuable for clinicians to make the prompt treatment decisions and avoid the unnecessary risks during a period of uncertainty. Researchers usually evaluate whether patients respond to ctDCS by patients’ seizure frequency reports during a period of time (at least 1 month) or monitoring the scalp electroencephalogram (EEG) after the stimulation, which may not be reliable enough, because patients often omit or forget the seizure attacks, and the interictal epileptiform discharges sometimes are not parallel to the clinical severity. So far, no objective and accurate markers have been found to predict the treatment outcome of ctDCS promptly after the stimulation.

The clinical, pathologic and imaging features have provided increasing evidence that epilepsy is a disorder that affects the brain network beyond the seizure onset zones (Gleichgerrcht et al., 2015). Many investigations have observed the abnormalities in the brain networks of patients with epilepsy using EEG, magnetoencephalogram (MEG), and functional magnetic resonance imaging (fMRI) data (Bettus et al., 2008; Luo et al., 2012; Pedersen et al., 2015; Wang and Meng, 2016). These abnormalities of brain networks are clinically relevant since they are capable of being important markers for the diagnosis and prediction of treatment outcome (Douw et al., 2010; van Diessen et al., 2013; van Dellen et al., 2014). Meanwhile, it has been increasingly apparent that tDCS modulates the brain network rather than just the stimulation targets (Luft et al., 2014; To et al., 2018). Based on EEG or fMRI, some studies have found that tDCS can modulate the functional connectivity of the brain networks in healthy subjects (Keeser et al., 2011; Polanía et al., 2011a, b; Vecchio et al., 2018). And for patients with epilepsy, Tecchio et al. (2018) and Lin et al. (2018) revealed the alterations of the functional connectivity after tDCS and the correlation between the functional connectivity and the seizure reduction. However, no studies have predicted the treatment outcome of ctDCS by investigating the functional network alterations so far. Thus, analyzing the ctDCS-induced alterations of the brain networks in patients with epilepsy to predict the treatment outcome of ctDCS is promising for clinical application.

Resting-state fMRI, with high spatial resolution and whole-brain coverage, has been increasingly utilized to investigate the functional connectivity of the brain network (Bernhardt et al., 2015). Based on fMRI, several studies have investigated the functional networks of patients with epilepsy employing seed-based functional connectivity analysis (Waites et al., 2006; Kim et al., 2014), or independent component analysis (ICA) (Zhang et al., 2009; Luo et al., 2012). However, these studies focused on the local abnormalities, which means only a few regions or some specific local networks were analyzed. In fact, the whole brain can be modeled as a graph consisting of nodes (regions) and edges (interregional connections) (Bullmore and Sporns, 2009). Recently, the whole-brain functional network in epilepsy has been increasingly investigated using graph theoretical analysis (Liao et al., 2010; Onias et al., 2014; Pedersen et al., 2015), which may provide further network-level information and improve our understanding about epilepsy.

In this study, the functional networks of patients with focal epilepsy were compared before and after the 5-day, −1.0 mA, 20-min ctDCS using graph theoretical analysis based on resting-state fMRI. By analyzing the ctDCS-induced functional network alterations, we aimed to find the markers to evaluate and predict the treatment outcome of ctDCS promptly after the stimulation.

## Materials and Methods

### Subjects

Twenty-three patients were recruited from the Department of Neurology of Zhongshan Hospital (Shanghai, China). The inclusion criteria included: (1) clinical diagnosis as focal epilepsy for at least 1 year according to epilepsy classification by International League Against Epilepsy (ILAE); (2) age between 18 and 65 years old; (3) at least one seizure at 4-week baseline; (4) stable anti-epileptic drug regimens from baseline to the 4-week follow-up; and (5) completion of 4-week follow-up after the treatment and ability to record the number of seizures. Exclusion criteria consisted of: (1) primary generalized epilepsy; (2) presence of status epilepticus during the last 12 weeks; (3) previous neuromodulation treatment such as tDCS, transcranial magnetic stimulation (TMS), vagus nerve stimulation (VNS), or deep brain stimulation (DBS) during the last 12 weeks; (4) implantation of pacemakers or other metal implants; and (5) other neural or systemic diseases, skull deficit, major depression or pregnancy.

Four patients suffering from primary generalized epilepsy were excluded from this study and the other 19 patients participated in this study. Among the 19 patients with focal epilepsy, eight patients underwent two ctDCS sessions with an interval of at least 12 weeks, resulting in 27 subjects. Written informed consents were obtained from the participants with a research protocol which was approved by the Ethics Committee of Zhongshan Hospital (Clinical Trial NCT02613234).

### Experimental Design

**FIGURE 1:**
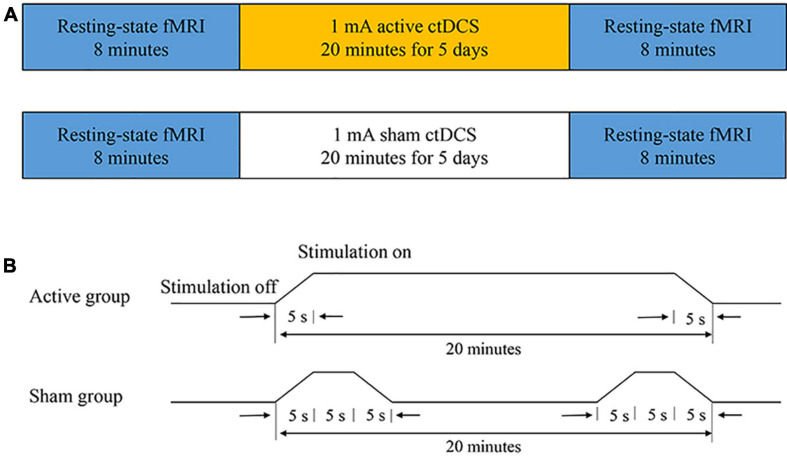
The experimental protocol. **(A)** The ctDCS-fMRI experimental procedure including active stimulation (top) and sham stimulation (bottom). **(B)** The ctDCS protocol for the active and the sham group, respectively.

The study was conducted in a randomized, double-blinded, sham-controlled design. All subjects were randomly divided into two groups: the active group and the sham group. Before the ctDCS treatment, the first 8-min fMRI scan was conducted for each subject. After a 5-day ctDCS treatment, the second 8-min fMRI scan was conducted (Figure 1A). Each subject was required to keep a seizure diary and record the number of seizures during the 4-week baseline and the 4-week follow-up. Neither the subjects nor the statistic analyzers knew the allocation of the trial.

So far, no studies have clearly defined the standard for whether patients respond to ctDCS in the treatment of epilepsy. Because of the placebo effect, the seizure frequency may reduce after the sham stimulation. Previous studies have reported −25.0, 11.1, and 6.25% reduction rate of seizure frequency in the sham treatment group, respectively (Fregni et al., 2006; Assenza et al., 2017; San-Juan et al., 2017). We supposed that the reduction rate of less than 20% may be due to the placebo effect. Thus, in this study, we selected 20% reduction rate as the threshold to eliminate the effect of placebo. Subject was considered to respond to ctDCS if the number of seizures during the 4-week follow-up was at least 20% less than the number of seizures during the 4-week baseline. Based on it, the subjects in the active group were then divided into the active group with response to ctDCS and the active group without response to ctDCS.

### Cathodal Transcranial Direct Current Stimulation

The ctDCS was administered using a BrainSTIM stimulator (EMS, Bologna, Italy). A direct current was applied to the scalp of the subject via a pair of saline-soaked sponge electrodes (area = 5 cm × 7 cm). The electrodes were placed according to the 10-10 international system. The cathode was placed over the most active region with interictal epileptiform discharges, confirmed by the 2-h EEG recording. The anode was positioned over a silent area without epileptogenic activity, which generally is the contralateral supraorbital region. All subjects underwent a 5-day ctDCS treatment. For the active group, a direct current of −1.0 mA was applied for 20 min each day (including a 5-s ramp-up at the beginning and a 5-s ramp-down at the end of the stimulation). If there were two epileptogenic foci for one subject, each site was stimulated for 10 min. For the sham group, a direct current of −1.0 mA was applied for 15 s (including a 5-s ramp-up and a 5-s ramp-down) at the beginning and the end of the 20-min course (Figure 1B). The subjects in the sham group had the same itching sensation as the subjects in the active group, and the subjects could not distinguish between the active ctDCS and the sham ctDCS.

### Functional Magnetic Resonance Imaging Acquisition

All subjects were scanned on a 3-Tesla scanner (GE-Signa, HDX3T, Milwaukee, Untied States) at the Zhongshan Hospital. Subjects were instructed to relax, keep their heads still and keep their eyes closed. Blood oxygen level dependent (BOLD) functional images were obtained using an EPI sequence (TR = 2,000 ms, TE = 30 ms, flip angle = 90°, matrix = 64 × 64, FOV = 22 cm × 22 cm, slice thickness = 4 mm, number of slices = 33, number of volumes = 240). For each scan, the fMRI scanning process lasted for 8 min with 240 time points.

### Functional Magnetic Resonance Imaging Preprocessing

All fMRI preprocessing steps were carried out using the DPARSF toolbox^1^ (Yan and Zang, 2010). The first ten volumes of each scan were discarded to allow for magnetization equilibrium. After the slice-timing correction, images were realigned for head-motion correction. The subjects were excluded if either translation or rotation of the head-motion in any direction exceeded ±2 mm or ±2°. Furthermore, the common Montreal Neurological Institute (MNI) template was used for normalization (resampling voxel size = 3 mm × 3 mm × 3 mm). The data were spatially smoothed using an isotropic Gaussian kernel with the FWHM of 8 mm and temporally band-pass filtered with the cutoff frequencies of 0.01 and 0.08 Hz. The global brain signals of the fMRI time series associated with the white matter signals, cerebrospinal fluid signals and six head-motion parameters were regressed out from the data (Fox et al., 2005).

### Anatomical Parcellation and fMRI Data Augmentation

The fMRI data were segmented into 90 (45 for each hemisphere) regions of interest (ROIs) using the automated anatomical labeling template (AAL) (Tzourio-Mazoyer et al., 2002). The mean time series of each region was obtained by averaging time series of all voxels in the region.

Extraction of the overlapping time windows is a common way to augment time-series data (Eslami et al., 2019). Since our sample size is small, we augmented the data by cropping the time series with 230 time points to length *T* = 90 with an interval of 35 time points and overlapping 55 time points. Thus, we obtained five time series of length *T* = 90 from the original time series. This process augmented the number of the samples by a factor of five (Dvornek et al., 2017; Li and Fan, 2019).

### Whole-Brain Network Construction

To measure the pair-wise functional connectivity among the regions and construct the whole-brain functional networks before and after ctDCS, the Pearson correlation coefficients between the time series of all pairs of regions were calculated for each sample. Then, the correlation coefficients were normalized using a Fisher’s R to Z transformation (Mudholkar, 2004). This step resulted in a 90 × 90 correlation matrix for each sample before and after ctDCS, respectively. Taking each brain region as a node and the interregional functional connection as an edge, the graph for each sample was constructed.

Finally, the graph was thresholded by a range of pre-defined density threshold values (the percentage of edges maintained in the network after thresholding) to obtain the undirected weighted networks with the same number of nodes and edges across subjects (van Wijk et al., 2010). To discard the negative connections of the networks and keep the networks connected, the density threshold values ranged from 0.17 to 0.46 with increments of 0.01 in this study. BrainNet Viewer was employed for the visualization of the networks^2^ (Xia et al., 2013).

### Graph Theoretical Analysis

Graph theoretical measures were calculated by Brain Connectivity Toolbox (BCT)^3^ implemented in MATLAB (R2019a, the Math Works, Natick, MA, United States) (Rubinov and Sporns, 2010). The definition of the measures we used in this study is provided in Table 1.

**TABLE 1:** The definition of graph theoretical measures.

Measure	Equation	Definition
Degree (local)	$K_{i}=\sum_{j\in G}w_{i\,j}$	The degree of node *i* is defined as the sum of the weights of edges connected with node *i*. *w*_*ij*_ is the connectivity strength between node *i* and node *j*
Clustering coefficient (local)	Cl⁢o⁢c⁢a⁢l,i=eiwKi⁢(Ki-1)/2	The clustering coefficient of node *i* is the measure of extent of interconnectivity among the nearest neighbors of node *i*. eiw is geometric mean of triangles around node *i*
Mean clustering coefficient (global)	$C_{n\,e\,t}=\frac{1}{N}\,\sum_{i\in G}C_{l\,o\,c\,a\,l,i}$	The mean clustering coefficient is the measure of network segregation, which is defined as the average of the clustering coefficients of all nodes in the network. *N* is the number of the nodes in the network
Mean shortest path length (local)	Ll⁢o⁢c⁢a⁢l,i=1N-1⁢∑j∈G,j≠idi⁢jw	The mean shortest path length of node *i* is the average of the shortest path lengths between node *i* and other nodes. di⁢jw is the shortest path length between node *i* and node *j*
Characteristic path length (global)	$L_{n\,e\,t}=\frac{1}{N}\,\sum_{i\in G}L_{l\,o\,c\,a\,l,i}$	The characteristic path length is the measure of global integration, which is defined as the average of shortest path lengths between all nodes in the network
Global efficiency (global)	En⁢e⁢t=1N⁢∑i∈G∑j≠i,j∈G(di⁢jw)-1N-1	The global efficiency is the measure of network’s ability for information transmission and is defined as the inverse of harmonic mean of shortest path lengths between any pair of nodes
Local efficiency (local)	*E*_*local*,*i*_ = *E*_*net*_(*G*_*i*_)	The local efficiency is the global efficiency computed on the neighborhood of the node. *G_i* denotes the subgraph composed of the neighbors of the node *i*
Small-worldness (global)	$\sigma =\frac{C_{n\,e\,t}/C_{R}}{L_{n\,e\,t}/L_{R}}$	Here, *C_R* and *L_R* are the averages of the mean clustering coefficients and the characteristic path lengths of 100 random networks, which are generated by randomly rewiring the edges while preserving the degree distribution of the original networks

In this study, the global network measures were assessed with the mean clustering coefficient (*C*_net_), the characteristic path length (*L*_net_), the global efficiency (*E*_net_), and the small-worldness (σ). When it comes to the local network measures related to the stimulation sites, the nodal degree (*K*), the clustering coefficient (*C*_local_), the mean shortest path length (*L*_local_), and the local efficiency (*E*_local_) were calculated. The stimulation sites were mapped to the corresponding brain regions in the AAL template by experienced clinicians. If there was only one corresponding node, the measures of the node were considered as the local network measures at the stimulation sites. Otherwise, the average measures of all corresponding nodes were calculated.

### Statistical Analysis

To determine if there were significant differences in the graph theoretical measures after ctDCS compared to those before ctDCS, statistical comparisons were performed over a range of density thresholds (from 0.17 to 0.46 with increments of 0.01). For each density threshold, if the differences of the measures before and after ctDCS were normally distributed, paired *t*-tests were performed. Otherwise, Wilcoxon signed-rank tests were employed. False discovery rate (FDR) was applied to *P*-values. *P*-values less than 0.05 after the FDR correction were considered significant.

### Treatment Outcome Prediction

The change rates of the measures which changed significantly after ctDCS for the active group with response to ctDCS but didn’t change significantly for the active group without response to ctDCS were used as the features to predict the treatment outcome of ctDCS. A two-step feature selection procedure was utilized to select the most informative features for the best prediction performance (Wei et al., 2019; Wen et al., 2019). In the first step, we calculated the correlations between every feature and class labels using the maximal information coefficient (MIC) and the features were ranked according to their MIC values (Reshef et al., 2011). In the second step, the sequential forward search (SFS) strategy was performed to search for the optimal feature subset which achieved the best performance in terms of the prediction accuracy (Theodoridis and Koutroumbas, 2009).

Support vector machine (SVM), a powerful machine learning algorithm (Cristianini and Shawe-Taylor, 2000), was employed for the treatment outcome prediction, which was implemented in the scikit-learn (v 0.22.1) library in Python. The radial basis function (RBF) kernel function was used in this study because of its best prediction performance when compared with the linear, polynomial and sigmoid. Besides, a nested cross validation strategy was used to evaluate the performance of the model. The dataset was randomly divided into five subsets with equal size. Four subsets were chosen as the training set and the other one subset was chosen as the testing set. This process was repeated five times with each subset used once as the testing set. And for the training set, a five-fold grid search cross validation strategy was used to select the optimal hyperparameters (C and γ) of the model, which means the training set was randomly divided into five subsets of which four subsets were training set and one subset was validation set each time. For the performance evaluation, three common measures were used, which were accuracy (ACC), sensitivity (SN), and specificity (SP) (Xu et al., 2012). Receiver operating characteristic (ROC) curve was employed to measure the overall performance of the model and the area under the ROC curve (AUC) was obtained (Fawcett, 2006).

## Results

### Group Division

Among 27 subjects, one subject was excluded because either translation or rotation of his or her head motion exceeded ±2 mm or ±2° during the fMRI data acquisition, and six subjects without valid clinical or fMRI follow-up were also excluded. Thus, a total of 20 subjects were included in this study (mean age: 39.8 ± 14.6 years, 11 females), among whom there were 12 subjects in the active group and eight subjects in the sham group. No significant differences were found in the general characteristics including sex, age and course of epilepsy between the active and the sham group (*P* > 0.05).

Clinical information is provided in Table 2. Four subjects responded to the active ctDCS since their reduction rates of seizure frequency were higher than 20% and eight subjects didn’t respond to the active ctDCS. After the data augmentation, the fMRI data of 20 subjects were augmented by a factor of five to a total of 100 samples. All samples were divided into three groups: (1) the active group with response to ctDCS (*n* = 20); (2) the active group without response to ctDCS (*n* = 40); and (3) the sham group (*n* = 40).

**TABLE 2:** Clinical information of the subjects.

Group	Subject	Sex	Age	Course (years)	Epileptic Discharge site	Cause	Seizure type	Epilepsy Syndrome	MRI Lesion	Cathode	Anode	Seizure frequency (baseline/follow-up) unit: times/4 weeks)	Response (Y/N)
Active	1^a^	M	30	17	Focal	Birth asphyxia and brain surgery	Focal clonic seizure	Epilepsy attributed to structural causes	Softening of left parietal lobe	Between C3-FC1	rSO^c^	80/77	N
	2^a^	M	27	2	Focal	Hippocampal sclerosis	Automatisms	Mesial temporal lobe epilepsy with hippocampal sclerosis	Hippocampal sclerosis	F7	rSO	1/4	N
	3^a^	M	54	40	Focal	Trauma	Focal onset to bilateral tonic-clonic seizure	Frontal lobe epilepsy	None	F4	P7	1/1	N
	4^a^	M	54	40	Focal	Trauma	Focal onset to bilateral tonic-clonic seizure	Frontal lobe epilepsy	None	F4	P7	1/3	N
	5	F	37	6	Focal	Gray matter heterotopia	Automatisms	Epilepsy attributed to heterotopia	Subependymal gray matter heterotopia	F7	rSO	3/1	Y
	6	F	41	2	Focal	Hippocampal sclerosis	Behavior arrest	Mesial temporal lobe epilepsy with hippocampal sclerosis	Hippocampal sclerosis	F7	rSO	10/14	N
	7	F	64	44	Focal	Hippocampal sclerosis	Automatisms, and focal onset to bilateral tonic-clonic seizure	Mesial temporal lobe epilepsy with hippocampal sclerosis	Hippocampal sclerosis	F8	lSO^d^	9/7	Y
	8^a^	F	23	23	Focal	Cryptogenic	Behavior arrest	Epilepsy of unknown cause	None	Between C3-F3	rSO	7/14	N
	9	F	28	10	Multifocal^b^	Viral encephalitis	Focal onset to bilateral tonic-clonic seizure	Epilepsy attributed to infection	Bilateral temporal lobe atrophy	F7/F8	rSO/lSO	12/9	Y
	10^a^	F	26	3	Multifocal^b^	Gray matter heterotopia	Automatisms	Epilepsy attributed to heterotopia	Subependymal gray matter heterotopia	F7/F8	rSO/lSO	5/10	N
	11	M	30	26	Focal	Focal cortical dysplasia	Focal onset to bilateral tonic-clonic seizure	Epilepsy attributed to focal cortical dysplasia	Focal cortical dysplasia in left frontal lobe	Between F4-Fz	P7	28/28	N
	12	M	64	10	Focal	Trauma	Focal onset to bilateral tonic-clonic seizure	Epilepsy attributed to trauma	Softening of left temporal lobe	F7	rSO	1/0	Y
Sham	13^a^	M	30	17	Focal	Hippocampal sclerosis	Automatisms	Mesial temporal lobe epilepsy with hippocampal sclerosis	Hippocampal sclerosis	Between C3-FC1	rSO	56/66	/
	14	M	47	16	Focal	Trauma and cavernous hemangioma	Automatisms, and focal onset to bilateral tonic-clonic seizure	Epilepsy attributed to structural causes	Cavernous hemangioma	CP6	lSO	2/2	/
	15^a^	M	27	2	Focal	Focal cortical dysplasia	Focal onset to bilateral tonic-clonic seizure	Epilepsy attributed to focal cortical dysplasia	Focal cortical dysplasia in left frontal lobe	F7	rSO	3/2	/
	16	F	61	53	Focal	Hippocampal sclerosis	Automatisms, and focal onset to bilateral tonic-clonic seizure	Mesial temporal lobe epilepsy with hippocampal sclerosis	Hippocampal sclerosis	F7	rSO	6/8	/
	17	F	49	14	Focal	Meningitis	Automatisms, and focal onset to bilateral tonic-clonic seizure	Epilepsy attributed to infection	White matter lesions	Between C4-P4	lSO	2/1	/
	18^a^	F	23	23	Focal	Cryptogenic	Behavior arrest	Epilepsy of unknown cause	None	Between C3-F3	rSO	14/9	/
	19	F	54	48	Focal	Poisoning and hippocampal sclerosis	Automatisms, and focal onset to bilateral tonic-clonic seizure	Mesial temporal lobe epilepsy with hippocampal sclerosis	Hippocampal sclerosis	F8	lSO	6/6	/
	20^a^	F	26	3	Multifocal^b^	Gray matter heterotopia	Automatisms	Epilepsy attributed to heterotopia	Subependymal gray matter heterotopia	F7/F8	rSO/lSO	6/4	/

*^a^These subjects underwent two ctDCS sessions with an interval of at least 12 weeks.*

*^b^These subjects suffered from multifocal epilepsy. Each site was stimulated for 10 min per day.*

*^c^rSO means the right supraorbital area.*

*^d^lSO means the left supraorbital area.*

### Functional Brain Network

**FIGURE 2:**
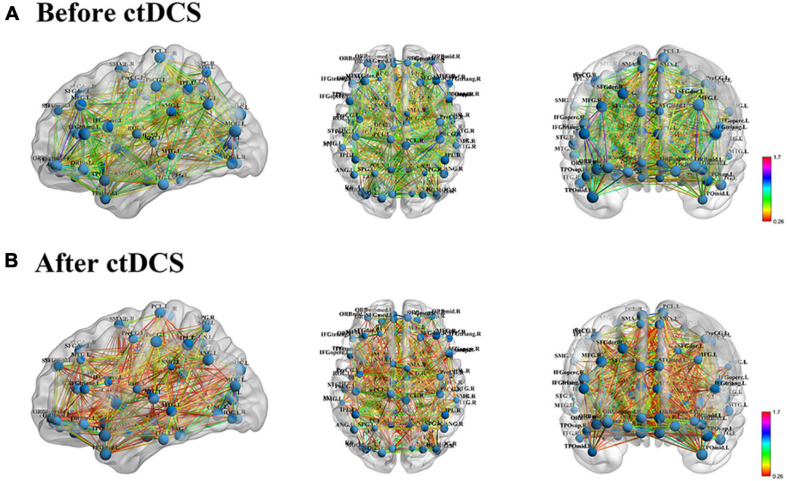
Functional brain network visualization for one sample of subject 7. **(A)** Functional brain network before ctDCS at the density threshold of 0.2 from three standard views, sagittal, axial and coronal. The size of the nodes represents the degree of the corresponding regions. The color of the edges represents the strength of the functional connectivity. **(B)** Functional brain network after ctDCS at the density threshold of 0.2.

Functional brain networks before and after ctDCS for each sample were constructed at the density threshold of 0.17–0.46 with an interval of 0.01. One example was visualized using BrainNet Viewer and shown in Figure 2. The brain networks were those before and after ctDCS at the density threshold of 0.2 for one sample of subject 7. As shown in Figure 2, for this sample, the strength of the functional connectivity decreased after ctDCS. To quantitatively analyze the network’s features, graph theoretical measures of the networks before and after ctDCS at different density thresholds were calculated and compared in the following sections.

### Global Network Measures

The global network measures were assessed with the mean clustering coefficient (*C*_net_), the characteristic path length (*L*_net_), the global efficiency (*E*_net_), and the small-worldness (σ). For the patients who underwent the active ctDCS, *C*_net_ decreased significantly after the stimulation (*P* < 0.01, FDR corrected), and the other measures did not change significantly.

**FIGURE 3:**
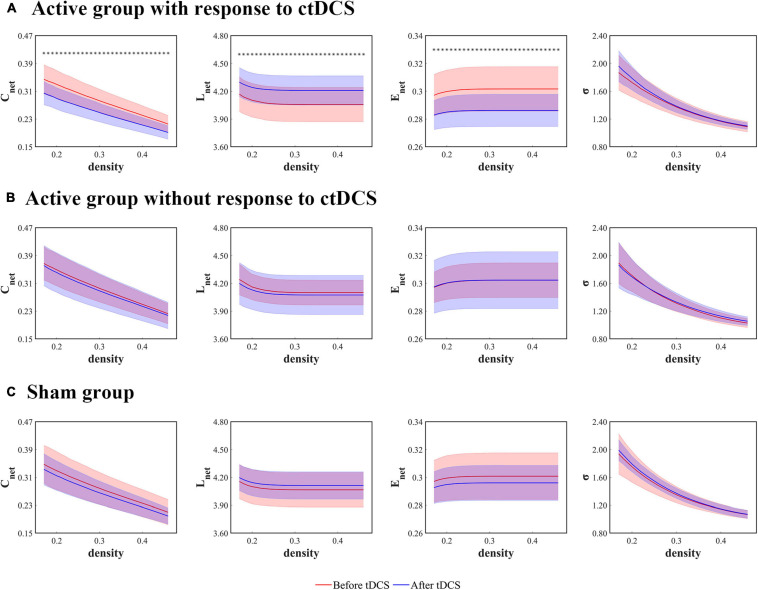
Global network measures as functions of network density thresholds before and after ctDCS. The global network measures include the mean clustering coefficient (*C*_net_), the characteristic path length (*L*_net_), the global efficiency (*E*_net_), and the small-worldness (σ) in the active group with response to ctDCS (*n* = 20) **(A)**, the active group without response to ctDCS (*n* = 40) **(B)**, and the sham group (*n* = 40) **(C)**. The solid lines indicate the mean values of the measures, and the shadows indicate the standard deviations of the measures. The red lines indicate measures before ctDCS and the blue lines indicate measures after ctDCS. The asterisks denote statistically significant differences before and after ctDCS (*P* < 0.01, FDR corrected).

When it comes to the ctDCS-induced alterations of network measures for the active group with response to ctDCS (Figure 3A), the active group without response to ctDCS (Figure 3B), as well as the sham group (Figure 3C), the statistical analysis found that within the whole range of density, there was a significant decrease of *C*_net_, a significant increase of *L*_net_, as well as a significant decrease of E_net_ after ctDCS for the active group with response to ctDCS (*P* < 0.01, FDR corrected), whereas for the active group without response to ctDCS and for the sham group, there were no significant differences in these measures after ctDCS (*P* > 0.05, FDR corrected). No significant differences were observed in σ before and after ctDCS for three groups (*P* > 0.05, FDR corrected).

### Local Network Measures

The local network measures were calculated using the degree (*K*), the clustering coefficient (*C*_local_), the mean shortest path length (*L*_local_), and the local efficiency (*E*_local_) at the stimulation sites. For the patients who underwent the active ctDCS, there were no significant differences in these measures before and after the stimulation.

**FIGURE 4:**
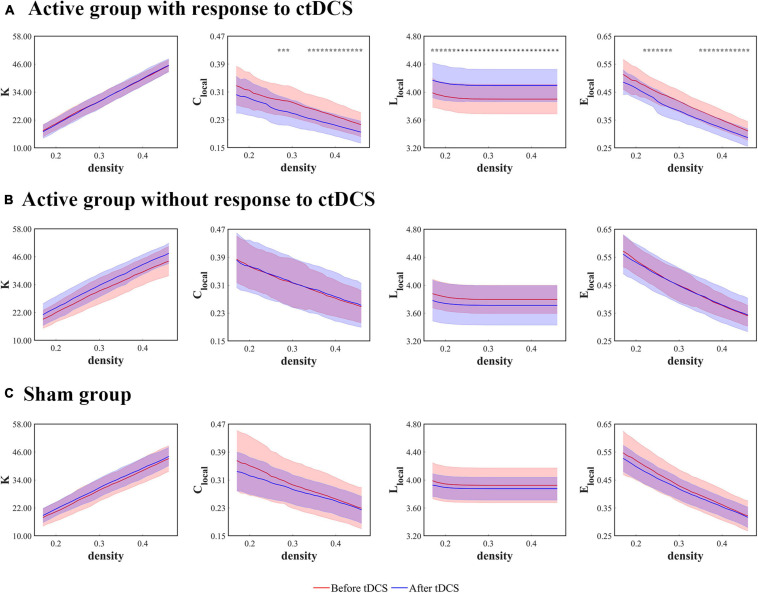
Local network measures as functions of network density thresholds before and after ctDCS. The local network measures include the degree (*K*), the clustering coefficient (*C*_local_), the mean shortest path length (*L*_local_), and the local efficiency (*E*_local_) at the stimulation sites in the active group with response to ctDCS (*n* = 20) **(A)**, the active group without response to ctDCS (*n* = 40) **(B)**, and the sham group (*n* = 40) **(C)**. The solid lines indicate the mean values of the measures, and the shadows indicate the standard deviations of the measures. The red lines indicate measures before ctDCS and the blue lines indicate measures after ctDCS. The asterisks denote statistically significant differences before and after ctDCS (*P* < 0.01, FDR corrected). The pentagrams denote statistically significant differences before and after ctDCS (0.01 < *P* < 0.05, FDR corrected).

When it comes to the ctDCS-induced alterations of the network measures for the active group with response to ctDCS (Figure 4A), the active group without response to ctDCS (Figure 4B), as well as the sham group (Figure 4C), the statistical analysis revealed that within a wide range of density, *L*_local_ at the stimulation sites increased significantly after ctDCS for the active group with response to ctDCS (*P* < 0.01, FDR corrected). No significant differences were found in K after ctDCS for the active group with response to ctDCS (*P* > 0.05, FDR corrected). As for *C*_local_ and *E*_local_, the significant decreases were found after ctDCS for the active group with response to ctDCS if *P* < 0.05 was considered significant. However, considering *P* < 0.01 significant, there were no significant differences. For the active group without response to ctDCS and the sham group, there were no significant differences in these measures after ctDCS (*P* > 0.05, FDR corrected).

### Treatment Outcome Prediction

As shown in Figures 3, 4, the measures, *C*_net_, *L*_net_, *E*_net_, and *L*_local_ at the stimulation sites, changed significantly after ctDCS for the active group with response to ctDCS but didn’t change significantly for the active group without response to ctDCS. In this study, the change rates of these four measures at the density threshold of 0.3 were used as the features to predict the treatment outcome of ctDCS. We ranked the features according to their MIC values and searched a subset of optimal features by the SFS strategy. We observed that when the features including the change rates of *C*_net_, *L*_net_, and *L*_local_ at the stimulation sites were selected, the model achieved the best performance.

**FIGURE 5:**
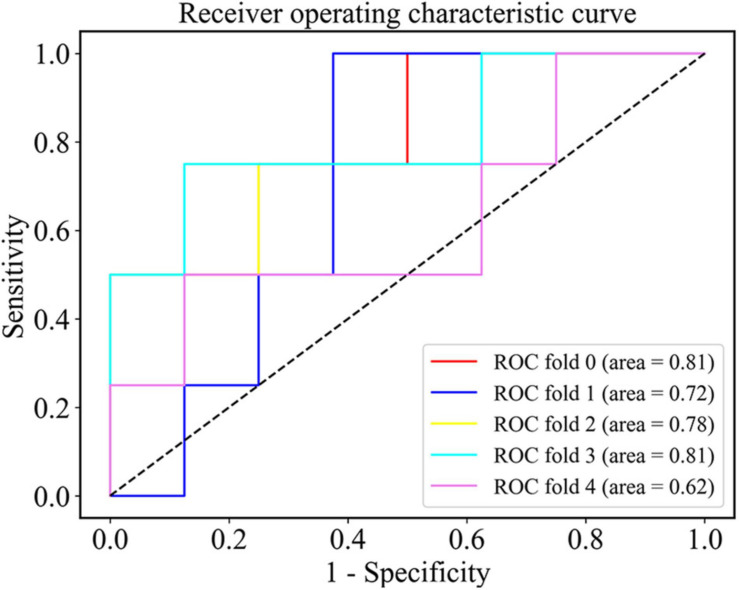
The ROC curve of the SVM classifier after the nested cross validation at the density threshold of 0.3.

After the nested cross validation, our model achieved the accuracy of 68.3 ± 11.1%, the sensitivity of 70.0 ± 18.7% and the specificity of 67.5 ± 24.5% at the density threshold of 0.3. The ROC curve after the nested cross validation is shown in Figure 5 with the area under the ROC curve (AUC) of 0.75 ± 0.07 at the density threshold of 0.3.

## Discussion

Transcranial direct current stimulation, as an emerging non-invasive neuromodulation technique, has been applied in the treatment of epilepsy in several studies (San-juan et al., 2015; Gschwind and Seeck, 2016). Studies *in vitro* and *in vivo* showed that ctDCS may modulate the excitability of the cortex, change the synaptic microenvironment, suppress the focal epileptiform discharges, and eventually restore the balance of the brain network (Purpura and McMurtry, 1965; Theodore and Fisher, 2007; Nune et al., 2015).

Small-sample studies reported ctDCS in focal epilepsy, in which the treatment outcomes were controversial (Varga et al., 2011; Auvichayapat et al., 2013; Assenza et al., 2014, 2017; Liu et al., 2016; Tekturk et al., 2016; San-Juan et al., 2017). It may be due to the different seizure types, the accuracy of the epileptogenic foci locating and the different therapy parameters. In our study, we found that not all of the patients benefited from the active ctDCS. Hence, it’s necessary in clinic to find out the potential group who can benefit from ctDCS using proper evaluation techniques, not delaying the treatment for those who will not benefit.

To our best knowledge, this study is the first to find that the response to ctDCS is related to the alterations of the functional network in epilepsy detected by fMRI. And the alterations of graph theoretical measures can serve as markers to predict the treatment outcome of ctDCS.

Several studies have reported that tDCS can modulate the functional networks (Keeser et al., 2011; Polanía et al., 2011a, b; Vecchio et al., 2018). For the patients with epilepsy, Tecchio et al. (2018) found that the functional connectivity changed after tDCS and the increase of the functional connectivity involving epileptic focus was correlated with seizure reduction based on EEG. Lin et al. (2018) observed that the phase lag index of alpha band decreased in the patients with seizure reduction after tDCS and increased in the patients without seizure reduction, which showed a negative correlation between the phase lag index and the seizure reduction. Based on these findings, we suppose that the alterations of functional networks after ctDCS may contribute to explain the response to ctDCS in the treatment of epilepsy.

An increasing number of studies have investigated the graph theoretical measures of functional networks in the patients with epilepsy compared to the healthy controls (Liao et al., 2010; van Dellen et al., 2012; Bartolomei et al., 2013; van Diessen et al., 2014). The results are conflicting, which may be due to the various seizure types and epileptogenic foci location, the different network construction methods and the different imaging modalities (Wang et al., 2014). Based on fMRI, EEG, or MEG, several studies reported an increase in the mean clustering coefficient (*C*_net_) (Vaessen et al., 2013; Wang et al., 2014; Wang and Meng, 2016), a decrease in the characteristic path length (*L*_net_) (Bartolomei et al., 2006; Liao et al., 2010), and an increase in the global efficiency (*E*_net_) (Doucet et al., 2015; Niso et al., 2015; Song et al., 2015) for the patients with epilepsy relative to the healthy controls. In this study, we found that for the patients with response to the active ctDCS, the decreased *C*_net_ was observed after ctDCS, indicating that the segregation of information processing reduced. Besides, for these patients, *L*_net_ increased, *E*_net_ decreased, and *L*_local_ at the stimulation sites increased after ctDCS, indicating the efficiency of propagating information reduced. We suppose that the alterations of the functional networks after ctDCS may make patients more prone to seizure reduction by reducing the local connectedness and the information transformation efficiency in the brain network.

The functional network alterations have been increasingly employed to provide clinically useful markers for the epilepsy diagnosis and the prediction of treatment outcome (Haneef and Chiang, 2014). van Diessen et al. (2013), Douw et al. (2010), and Zhang et al. (2012) constructed functional networks and diagnosed epilepsy using the random forest classifier, logistic regression analysis and SVM classifier, respectively. Douw et al. (2008) and van Dellen et al. (2014) compared the brain networks before and after the surgical resection and found that the surgical resection altered the brain networks in patients who were seizure-free after the treatment, which was promising for the prediction of treatment outcome. However, so far, no studies have predicted the treatment outcome of ctDCS by investigating the functional network alterations. We built an SVM prediction model based on fMRI which showed good performance, and the graph theoretical measures of functional networks, *C*_net_, *L*_net_, and *L*_local_ at the stimulation sites were proven to be markers with highly predictive power.

The present study has some limitations. Firstly, in this study, we augmented the data since the sample size is rather small, which may lead to data leakage. Larger studies are still needed to explore the actual predictive power of our model. In addition, the seizure types, the location, and the extent of epileptogenic zone of the patients participated in this study were heterogeneous. Future studies should consider more homogeneous patients to control the effects of these factors. Thirdly, in our study, ctDCS didn’t lead to a significant decrease in seizure frequency, which may result from the setting of the stimulation parameters. Future studies should explore different stimulation parameters, including the stimulation duration, current intensity, repeated sessions and so on (Yang et al., 2020). Fourthly, we selected the 20% reduction rate of seizure frequency after 4 weeks as the threshold for response to ctDCS to eliminate the placebo effect. Higher thresholds will be employed in future studies when the sample size increases. Fifthly, during the whole study, patients were still taking the anti-epileptic drugs. However, the interaction between the tDCS and the drugs were not analyzed.

## Conclusion

This study revealed the significant ctDCS-induced alterations of the graph theoretical measures only for the patients with response to the active ctDCS, indicating that response to ctDCS was related to the functional network alterations for the patients with epilepsy. Employing the changes of these graph theoretical measures as the inputs, we built an SVM prediction model to predict the treatment outcome of ctDCS with good performance. Our study demonstrated that the functional network alterations were promising to be the markers of predicting the treatment outcome of ctDCS.

## Data Availability Statement

The original contributions presented in the study are included in the article/supplementary material, further inquiries can be directed to the corresponding authors.

## Ethics Statement

The studies involving human participants were reviewed and approved by the Ethics Committee of Zhongshan Hospital. The patients/participants provided their written informed consent to participate in this study.

## Author Contributions

WL, YF, WP, and JD designed the study. WS acquired the data. JH, YX, and PZ analyzed the data. JH wrote the manuscript, while JZ, PZ, JD, and XW revised the manuscript. PZ and JD contributed to the funding acquisition. All authors contributed to the article and approved the submitted version.

## Conflict of Interest

The authors declare that the research was conducted in the absence of any commercial or financial relationships that could be construed as a potential conflict of interest.

## Footnotes

1 http://rfmri.org/DPARSF

2 https://www.nitrc.org/projects/bnv

3 http://www.brain-connectivity-toolbox.net
